# Supplementary figures and images for: Deep Dive into the Long Haul: Analysis of Symptom Clusters and Risk Factors for Post-Acute Sequelae of COVID-19 to Inform Clinical Care
Source: Int J Environ Res Public Health. 2022 Dec 15;19(24):16841. doi: 10.3390/ijerph192416841 (PMC9778884; doi:10.3390/ijerph192416841)

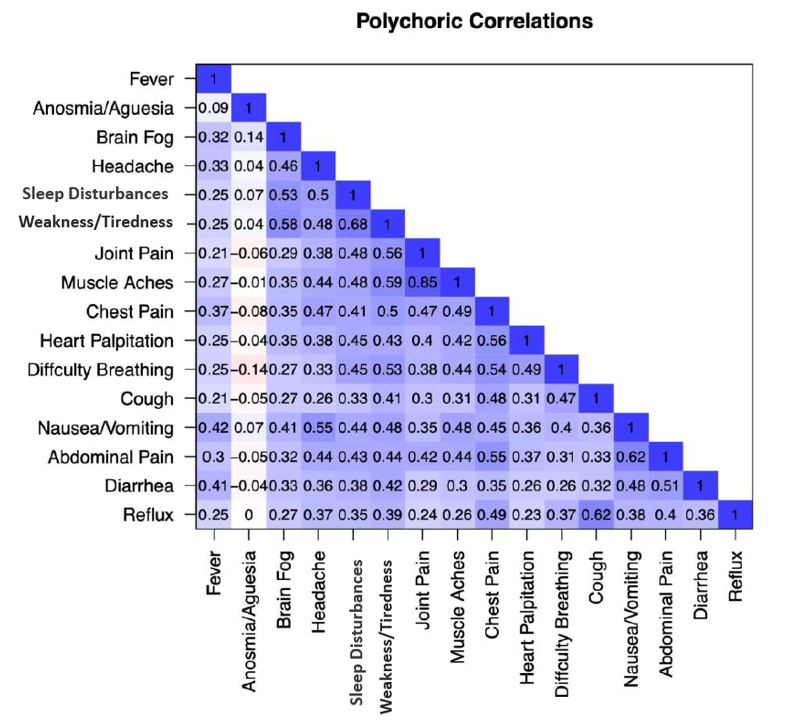

Supplement: Supplementary file 1 [file ijerph-19-16841-s001.zip › image_2022_12_15T07_54_14_453Z.png]
